# Generational IQ test score changes and the positive manifold of intelligence: evidence from Austrian Air Force pilots and air traffic controllers (1992–2016)

**DOI:** 10.3389/fpsyg.2025.1547520

**Published:** 2025-05-14

**Authors:** Sandra Oberleiter, Jana Wurzer, Michael Mikas, Martin Held, Bettina Wieland, Elisabeth L. Zeilinger, Martin Voracek, Jakob Pietschnig

**Affiliations:** ^1^Department of Developmental and Educational Psychology, Faculty of Psychology, University of Vienna, Vienna, Austria; ^2^Vienna Doctoral School in Cognition, Behavior, and Neuroscience (VDS CoBeNe), University of Vienna, Vienna, Austria; ^3^Department of Aviation Psychology, Austrian Armed Forces, Vienna, Austria; ^4^Department of Clinical Research SBG, Academy for Ageing Research, Haus der Barmherzigkeit, Vienna, Austria; ^5^Department of Clinical and Health Psychology, Faculty of Psychology, University of Vienna, Vienna, Austria; ^6^Department of Cognition, Emotion, and Methods in Psychology, Faculty of Psychology, University of Vienna, Vienna, Austria; ^7^Social Sciences Advisory Board, Science Commission at the Federal Ministry of Defence, Vienna, Austria

**Keywords:** Flynn effect, positive manifold of intelligence, psychometric g, Cattell-horn-Carroll (CHC) intelligence model, Austria, armed forces, pilots

## Abstract

Increasingly inconsistent generational IQ test score change patterns across recent decades have been suggested to be due to increased ability differentiation as a consequence of cross-temporally decreasing strengths of the positive manifold of intelligence. Here, we investigate the Flynn effect and directly test the idea of a changing positive manifold, based on the performance of *N* = 204 Austrian Air Force pilots and air traffic controllers across twelve IQ subtests. Subscale change scores indicated consistent gains in measures related to the CHC (Cattell-Horn-Carroll) stratum II domains of fluid reasoning, quantitative knowledge, and retrieval. However, change patterns in the stratum II domains working memory and comprehension knowledge were ambiguous and suggest stratum I-based differentiation of the Flynn effect. In all, our results indicate positive and substantial Flynn effects in the majority of examined subscales, but no evidence for any meaningful changes in the positive manifold strength.

## 1 Introduction

Cross-temporal IQ test score gains in the general population, known as the Flynn effect, were first systematically investigated in the 1980s (Flynn, 1984, 1987). Subsequent formal meta-analyses revealed that these trends were consistent throughout most of the 20th century, although they varied in strength across countries, cognitive subdomains, as well as ability segments (Pietschnig and Voracek, 2015; Trahan et al., 2014). On average, IQ test score increases have been estimated to amount to three points per decade for fullscale IQ, three to four points for fluid intelligence, and two points for crystallized intelligence (Pietschnig and Voracek, 2015).

Although Flynn effect change trajectories have been globally nonlinear from the very outset of the available evidence in the early 1900s, there is some evidence for a decreasing strength of gains since the 1980s (Pietschnig and Voracek, 2015). Interestingly, more recent studies suggest that Flynn effect patterns have become increasingly differentiated across countries and intelligence subdomains (Lazaridis et al., 2022; Pietschnig and Gittler, 2015). In fact, recent observations have shown stagnating and declining IQ test scores in several countries, particularly in Scandinavia (Denmark: Teasdale and Owen, 2008; Finland: Dutton and Lynn, 2013; Netherlands: Woodley and Meisenberg, 2013), but also in other countries (USA: Dworak et al., 2023).

One possible explanation for such a decreasing strength of gains or stagnations is that IQ-enhancing factors may already have reached their ceiling or may yield diminishing returns (e.g., perinatal nutrition, medical care; Pietschnig and Voracek, 2015; Pietschnig et al., 2018). This idea is consistent with the observation that the countries that were among the first to show a negative Flynn effect, such as Finland (Dutton and Lynn, 2013) and Denmark (Teasdale and Owen, 2008), are among the wealthiest nations globally where IQ-boosting environmental effects may have already peaked (Dutton et al., 2016).

Another possibility is that more fine-grained cognitive ability assessments due to the increasing sophistication and complexity of intelligence models, such as the now widely accepted Cattell-Horn-Carroll (CHC) theory of intelligence (Schneider and McGrew, 2018), may have contributed to the recently observed inconsistencies in IQ trajectories. Specifically, examinations of CHC-based stratum II domains beyond the classical taxonomy of fluid and crystallized intelligence sensu Cattell (1963) have revealed a considerable differentiation of domain trajectories. While some domains showed ability increases (e.g., attention: Andrzejewski et al., 2024), others revealed stagnations (e.g., reading comprehension: Lazaridis et al., 2022), ambiguous trends (e.g., visualization: Lazaridis et al., 2022), or even declines (e.g., induction: Oberleiter et al., 2024b).

These inconsistent trends may be attributable to the more elaborate cognitive ability assessments of more recent studies that allowed for observing previously under examined domain-specific test score changes (Oberleiter et al., 2024b). If the increased domain-specificity is indeed due to genuine differences in stratum II or even stratum I trajectories, one would expect that, when investigating cross-temporal test score changes on the subtest level of intelligence assessments, a differentiated pattern of gains, stagnation, and declines will emerge.

However, another recent account showed exclusively positive Flynn effects in all (measurement invariant) subtest scores of an intelligence test battery, whereas a simultaneous decrease in the strength of the intercorrelations among these subtests (i.e., the positive manifold of intelligence; see Oberleiter et al., 2024a; Andrzejewski et al., 2025), thus suggesting increasing ability differentiation as a potent driver of less consistent Flynn effects. Conceivably, the recently observed inconsistent change patterns in the Flynn effect may be attributed to a declining strength of the positive manifold of intelligence over time (Oberleiter et al., 2024a).

This assumption would be in line with well-established findings of negative correlations between the Flynn effect and psychometric *g* (e.g., Must et al., 2003; Pietschnig and Voracek, 2015; te Nijenhuis and van der Flier, 2013; Woodley and Madison, 2013), thus indicating that observed IQ gains are not due to improvements in the general factor of intelligence, but rather due to improvements in specific cognitive abilities (Pietschnig, 2021; Pietschnig et al., 2023).

If the positive manifold were observed to decrease over time, the observed change patterns of the Flynn effect could be driven by increasing differentiation in cognitive ability profiles within the general population over the past decades. One can think about this idea in terms of a decathlon (see Pietschnig, 2021, pp. 132–133; Oberleiter et al., 2024a), as illustrated in the following.

Decathletes receive points for their performance in ten specific athletic disciplines, the sum of which represents their overall performance. Nonetheless, the performances in different disciplines correlate, meaning that if someone excels in one discipline, they typically also excel in the other disciplines (i.e., they are a good athlete) and vice versa. In analogy to the positive manifold of intelligence, the overall decathlon performance can be thought of in terms of an athletics “*g*.”

If a decathlete were to focus their training on a specific discipline, their decathlon score would initially improve, but the correlation between different discipline performances would weaken. However, decathlon score improvements would only occur as long as the score increases in the trained discipline are larger than the sum of the incremental losses in the other (non-focused-on) disciplines. Were the decathlete to continue focusing on a single discipline, the gained performance increments would, inevitably so, become smaller over time due to Spearman’s law of diminishing returns (SLODR) (Spearman, 1927). This development will eventually yield stagnation of overall decathlon scores and, subsequently, a reversal of the overall performance trajectory.

This does not mean that this decathlete has become less of an athlete, but rather that he has become a specialist in a single discipline, as opposed to a generalist across many athletic disciplines. Overall, their “athletic *g*” may have decreased, but they definitely have become a more specialized runner, a more able hurdler, or a more successful javelin thrower (Pietschnig et al., 2023). Importantly, as a consequence of such a specialization, the performance in a specific discipline does not predict the performance in other disciplines as strongly as before, due to the decreased intercorrelations between the specific disciplines (in other words, a decreased strength in the positive manifold). Cognitive ability changes can be thought of in a similar manner if one assumes that specialization (i.e., ability differentiation) foremost occurs between generations, rather than within the lifespan of individuals.

It seems plausible that cognitive ability differentiation has become increasingly important in modern environments. Arguably, success in work or life in general nowadays requires more specific abilities, skills, and knowledge, thus incidentally reinforcing differentiation of abilities in the cognitive domain. In other words, the set of abilities and skills required in the modern world are likely to differ from those several decades (or just a few generations) ago, with contemporary environments reinforcing specialization instead of being a generalist.

Although the development and differentiation of cognitive abilities across successive generations may well occur in a more inconspicuous fashion compared to the acquisition of expertise, the mechanism driving this differentiation may be assumed to work in a similar manner. Therefore, ability differentiation could be responsible for *g* changes and increasingly domain-specific and inconsistent IQ trajectories (Pietschnig et al., 2023). Consistent with this idea, decreasing test intercorrelations have been observed in France during times of IQ score increases (Lynn and Cooper, 1993), and evidence from Estonian cohort data and Italian large-scale student assessments have provided tentative evidence for a link between ability differentiation and the Flynn effect (Woodley and Madison, 2013) and a decreasing strength of the positive manifold in achievement *g* (Pietschnig et al., 2023).

Moreover, recent evidence provides direct support for this argument (Oberleiter et al., 2024a; Andrzejewski et al., 2025). Analyses of population-representative Austrian samples using measurement-invariant subtests from an intelligence battery spanning up to 19 years have revealed test score gains across all subtests whilst at the same time showing statistically significant decreases of up to 7 % in the strength of the positive manifold (Oberleiter et al., 2024a). This indicates a stronger ability differentiation within the general population over the past few decades, possibly owing to an ongoing increase in the cognitive specialization of individuals.

Interestingly, these findings not only suggest a weakening of the intercorrelations between subtests and, therefore, a substantial decline in the positive manifold strength, but also a reduction in ability variance. Prior related research found ability increases to be more pronounced at the lower end of the ability distribution compared to the upper end, thus yielding IQ gains that were nearly twice as large (Lynn and Hampson, 1986; Pietschnig et al., 2013; Trahan et al., 2014; Flynn and Shayer, 2018; Oberleiter et al., 2024a). This suggests that the Flynn effect may be linked to a reduction in population variability instead of a continuous shift of the IQ distribution (Rodgers, 1998). A cross-temporal narrowing of the ability distribution, and consequently, restricted variances, could also contribute to the observed decline in the strength of the positive manifold. According to SLODR, cognitive abilities of individuals from the upper half of the ability distribution can already be assumed to be comparatively substantially differentiated, with psychometric *g* playing a subordinate role in their performance compared to individuals performing below the average (Spearman, 1927; Deary et al., 1996). Thus, due to variance restriction and SLODR, it can be hypothesized that high performers (i.e., beyond the average) may show less of a cross-temporal decline in the positive manifold of intelligence compared to low-performers (i.e., below the average) or the general population at large.

Here, we present the first targeted investigation of changes in the positive manifold of intelligence over time in a presumably highly specialized sample of Austrian Air Force pilots and air traffic controllers alongside cross-temporal changes in IQ test scores. To this end, we analyze a sample of *N* = 204, spanning a period of up to 25 years.

## 2 Methods

All Supplementary materials and the complete analysis code for this study are openly available on the Open Science Framework (OSF) at https://osf.io/2gxwa/.

### 2.1 Participants

We analyzed archival intelligence test data from initial aptitude assessments of *N* = 204 Austrian Air Force pilots (*n* = 133, 65.2%) and air traffic controllers (*n* = 69, 33.8%) from 1992 to 2016 (97.1% male, mean age = 21.3 *SD* = 3.8, age range = 18 to 37 years, see Table 1 for details). The participants underwent a highly selective and standardized assessment procedure conducted by the Austrian Armed Forces, targeting individuals for military aviation services. The selection process for Austrian Air Force pilots and air traffic controllers ensures that candidates fulfill mandatory standards regarding skills and aptitude for aviation roles, including overall (i.e., not at the subtest level) above-average cognitive abilities and technical skills. Furthermore, they passed rigorous psychological, physical, and medical evaluations. Only participants who successfully met these criteria were included in this study. On average, across all subtests, participants performed 0.51 standard deviation units (range: −0.73 to 1.42 *SD*s) above the population mean. Detailed descriptive statistics, including means and standard deviations for each subtest, are presented in Table 2.

**Table 1:** Sociodemographic sample characteristics.

Characteristics	*n*	%
Sex		
Male	198	97.1
Female	6	2.9
Educational level		
Compulsory education	2	1.0
Vocational middle school^a^	29	14.2
Post-secondary education^b^	171	83.8
University degree	1	0.5
Aviation role		
Air Force pilot	133	65.2
Air traffic controller	69	33.8

*N* = 204; For one participant, the highest educational level was unknown, and the aviation roles were also unknown for two male participants.

a Equivalent to level three according to the International Standard Classification of Education (ISCED) (UNESCO Institute for Statistics, 2012).

b Qualification for university admission.

**Table 2:** Means and standard deviations according to WIT subtests.

Subtest (stratum II domain)	*N*	*M*	*SD*	Distance to population
Digit span (*g*_wm_)	204	104.46	13.65	0.30
Letter sequencing (*g*_f_)	184	114.57	9.97	0.97
(medium-term) Memory (*g*_wm_)	184	119.08	12.25	1.27
Number sequencing (*g*_q_)	180	110.57	13.25	0.70
Numerical reasoning (*g*_q_)	180	96.28	12.07	−0.25
Observation (*g*_f_)	183	95.32	11.21	−0.31
Processing speed and spatial reasoning (*g*_f_)	167	119.99	12.50	1.33
Proverb comprehension (*g*_c_)	180	98.12	8.16	−0.13
Semantic similarities (*g*_c_)	180	104.95	8.99	0.33
Verbal analogies (*g*_c_)	204	121.33	10.38	1.42
Verbal arithmetic tasks (*g*_f_)	171	118.26	18.49	1.22
Verbal fluency (*g*_r_)	204	89.07	10.77	−0.73

Stratum II subdomains according to Schneider and McGrew (2018): g_c_, comprehension knowledge; g_f_, fluid reasoning; g_q_, quantitative knowledge; g_r_, retrieval fluency; g_wm_, working memory; distance to population = performance difference in SD units above (positive sign) or below (negative sign) the population mean.

### 2.2 Materials

Intelligence was assessed by means of the Wilde Intelligence Test (WIT) (Jäger and Althoff, 1983). The WIT is a well-established, comprehensive intelligence test battery comprising 15 subscales. It was developed for use in Germanophone adolescent and adult populations aged 14 to 38 years, and it has a test duration of approximately 4 h. In the data collection underlying this study, participants completed either twelve, ten, or three WIT subscales, depending on their recruitment year (i.e., during the period 1992 to 2016). The subscales administered here included digit span, letter sequencing, medium-term memory, number sequencing, numerical reasoning, observation, processing speed and spatial reasoning, proverb comprehension, semantic similarities, verbal analogies, verbal arithmetic tasks, and verbal fluency, thus mapping on the CHC-based stratum II domains *g*_c_, *g*_f_, *g*_q_, *g*_r_, and *g*_wm_ (see below). In our analyses, we included only participants who had completed at least three subscales. A detailed description of each subscale is available at https://osf.io/vcn59/.

### 2.3 Analysis

#### 2.3.1 Cross-temporal IQ test score changes

We conducted linear regression analyses to examine average IQ changes over time (the Flynn effect), i.e., predicting IQ scores based on data collection years (1992 to 2016). Given that the number of completed subscales varied by administration year, we ran separate analyses for datasets with twelve, ten, or three subscales. This approach allowed us to calculate regression slopes representing annual IQ changes for each subset, thereby covering twelve subscales for a period of 16 years (1992 to 2007), ten subscales for 21 years (1992 to 2012), and three subscales for 25 years (1992 to 2016).

#### 2.3.2 Cross-temporal changes of the positive manifold

To investigate possible changes in the strength of the positive manifold of intelligence over time, we performed a factor analysis for each of the three subsets of recruitment years (namely, the periods 1992 to 2007, 1992 to 2012, and 1992 to 2016). Following the approach of Oberleiter et al. (2024a), we used the respective available subscale scores of the WIT for each sample (i.e., twelve, ten, and three subscales, respectively). The explained variance was derived for each subset from a forced single-factor analysis (*R*^2^; this can be readily interpreted as an indicator for the strength of the positive manifold of intelligence), with subtest factor loadings reflecting the *g* loadings of the positive manifold. Furthermore, to assess cross-temporal changes in the *g* saturation of the WIT, we compared McDonald’s ω_h_ (omega hierarchical; see McDonald, 1999) within each subset of recruitment years. In contrast to other reliability indices or internal consistency measures, such as Cronbach’s coefficient α, ω_h_ specifically accounts for the reliability attributable to a general factor (here, psychometric *g*) in subtest scores. It reflects the *g* saturation of a test and is calculated as the ratio of the variance explained by the general factor compared to the total variance in the observed correlations (see Revelle and Wilt, 2013).

However, it is well-known that the stability of factor-analytic results is highly dependent on the available sample size, with factor solutions becoming increasingly unreliable as a function of decreasing sample size (see MacCallum et al., 1999). To examine whether potential changes in *R*^2^ and ω_h_ are merely a consequence of low case numbers or not, we ran cross-temporal cumulative “forward” and “backward” factor analyses and ω_h_ calculations within our data subsets of twelve, ten, and three subtests, starting or ending with years that included at least ten participants. If observed changes in the positive manifold remain directionally consistent in analyses with larger sample sizes (i.e., regardless of whether they originate from forwards or backwards cumulations), it can be assumed that any observed changes reflect genuine trends over time rather than artifacts arising from varying sample sizes.

Finally, we predicted changes in the within-participant difference between the maximum and minimum subtest IQ scores (i.e., individuals’ ability range) by recruitment years as an indicator for changes in ability differentiation. This is reasonable because the average IQ profile range can be interpreted as an indicator of the asymmetry of a given intelligence profile (i.e., the positive manifold strength), with larger ranges indicating larger asymmetries (i.e., indicating lower *g* saturation) and smaller ranges indicating less asymmetry (i.e., indicating larger *g* saturation).

All analyses were conducted using R 4.4.2 (R Core Team, 2024) and RStudio 2024.09.1 + 394 (RStudio Team, 2024), using the R packages lavaan (Rosseel, 2012) and psych (Revelle, 2024).

## 3 Results

### 3.1 Cross-temporal IQ test score changes

First, our analyses of average IQ changes revealed consistent ability increases (i.e., positive Flynn effects) from 1992 to 2007 across all twelve subtests. Annual gains ranged from 0.39 IQ points in letter sequencing up to 2.37 IQ points in verbal arithmetic tasks (*p* range: < 0.001 to 0.59).

Second, IQ trajectories from 1992 to 2012 showed a largely consistent pattern of ability increases across nine of the ten subtests available for this analysis. These positive Flynn effects ranged from annual gains of 0.09 IQ points in semantic similarities up to 0.86 IQ points in (medium-term) memory (*p* range: < 0.001 to 0.51). However, for verbal analogies, we observed a negative Flynn effect, with an annual decrease of −0.06 IQ points (*p* = 0.66).

**Figure 1:**
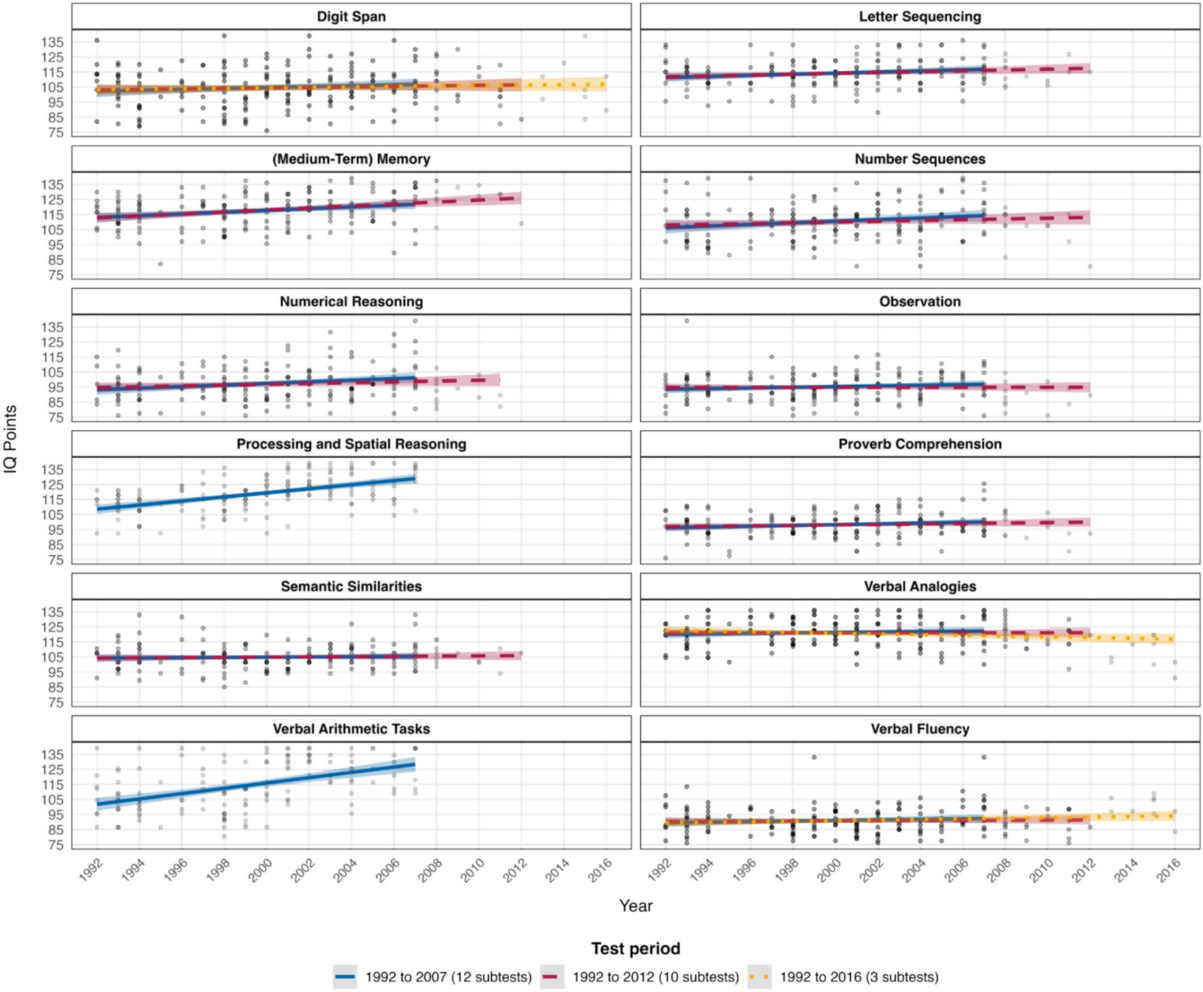
IQ subscale changes over time for the respective test periods from 1992 to 2016. As a robustness check, we reran our analyses by excluding participants with IQs > 130 for the verbal fluency and observation subscales. Results were virtually identical and indicated no substantial changes in the parameter estimates of the regression models.

Third, analyses revealed an inconsistent pattern of IQ trajectories across the three subtests with data available from 1992 to 2016. Positive Flynn effects were observed in verbal fluency and digit span, with annual gains yielding 0.32 IQ points (*p* = 0.01) and 0.16 IQ points (*p* = 0.31), respectively. Interestingly, verbal analogies showed a negative Flynn effect, with an annual decrease of −0.30 IQ points (*p* = 0.02). Numerical details are provided in Table 3 and Figure 1.

**Table 3:** Annual IQ point changes (and changes per decade) according to WIT subtests.

Subtest (stratum II domain)	1992–2007 *N* = 167–172	1992–2012 *N* = 180–195	1992–2016 *N* = 204
Digit span (*g*_wm_)	0.305 (3.05)	0.180 (1.80)	0.165 (1.65)
Letter sequencing (*g*_f_)	0.391 (3.91)*	0.298 (2.98)*	-
(medium-term) Memory (*g*_wm_)	0.842 (8.42)**	0.862 (8.62)**	-
Number sequencing (*g*_q_)	0.690 (6.90)**	0.369 (3.69)	-
Numerical reasoning (*g*_q_)	0.482 (4.82)*	0.174 (1.74)	-
Observation (*g*_f_)	0.427 (4.27)*	0.154 (1.54)	-
Processing speed and spatial reasoning (*g*_f_)	1.396 (13.96)**	-	-
Proverb comprehension (*g*_c_)	0.256 (2.56)	0.154 (1.54)	-
Semantic similarities (*g*_c_)	0.089 (0.89)	0.089 (0.89)	-
Verbal analogies (*g*_c_)	0.094 (0.94)	−0.060 (−0.60)	−0.297 (−2.97)*
Verbal arithmetic tasks (*g*_f_)	2.371 (23.71)**	-	-
Verbal fluency (*g*_r_)	0.262 (2.62)	0.181 (1.81)	0.319 (3.19)*

Stratum II subdomains according to Schneider and McGrew (2018): g_c_, comprehension knowledge; g_f_, fluid reasoning; g_q_, quantitative knowledge; g_r_, retrieval fluency; g_wm_, working memory; *p < 0.05; **p < 0.01.

### 3.2 Cross-temporal changes of the positive manifold

**Figure 2:**
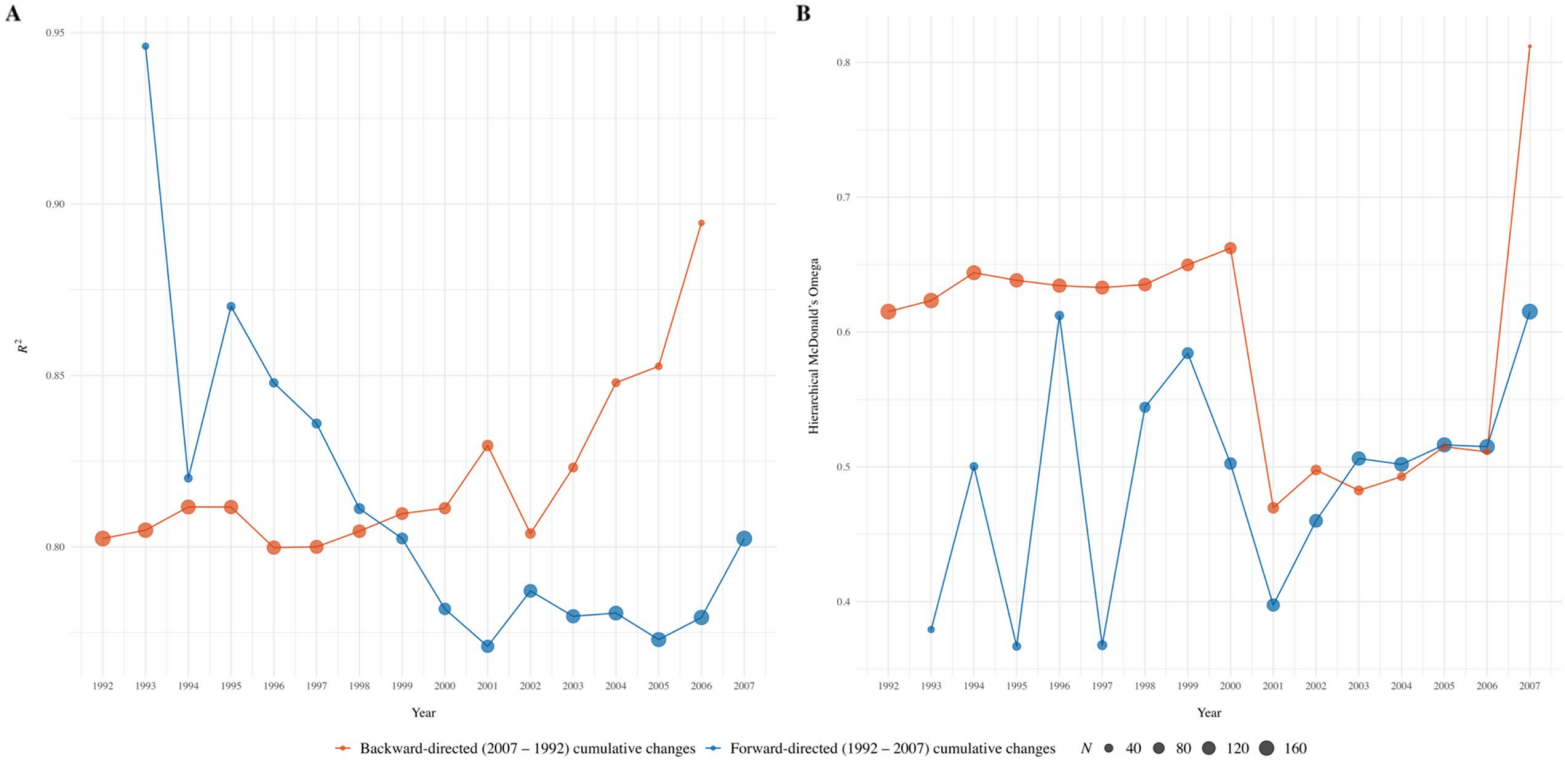
Forward-directed (1992 to 2007) and backward-directed (2007 to 1992) cumulative cross-temporal changes in *R*^2^ **(Panel A)** and McDonald’s ω_h_ **(Panel B)** across twelve WIT subscales. The cumulation of those years that contained data from at least ten participants was chosen as the starting point; within backward-directed factor analyses for 2007, the model did not converge due to low case numbers.

**Figure 3:**
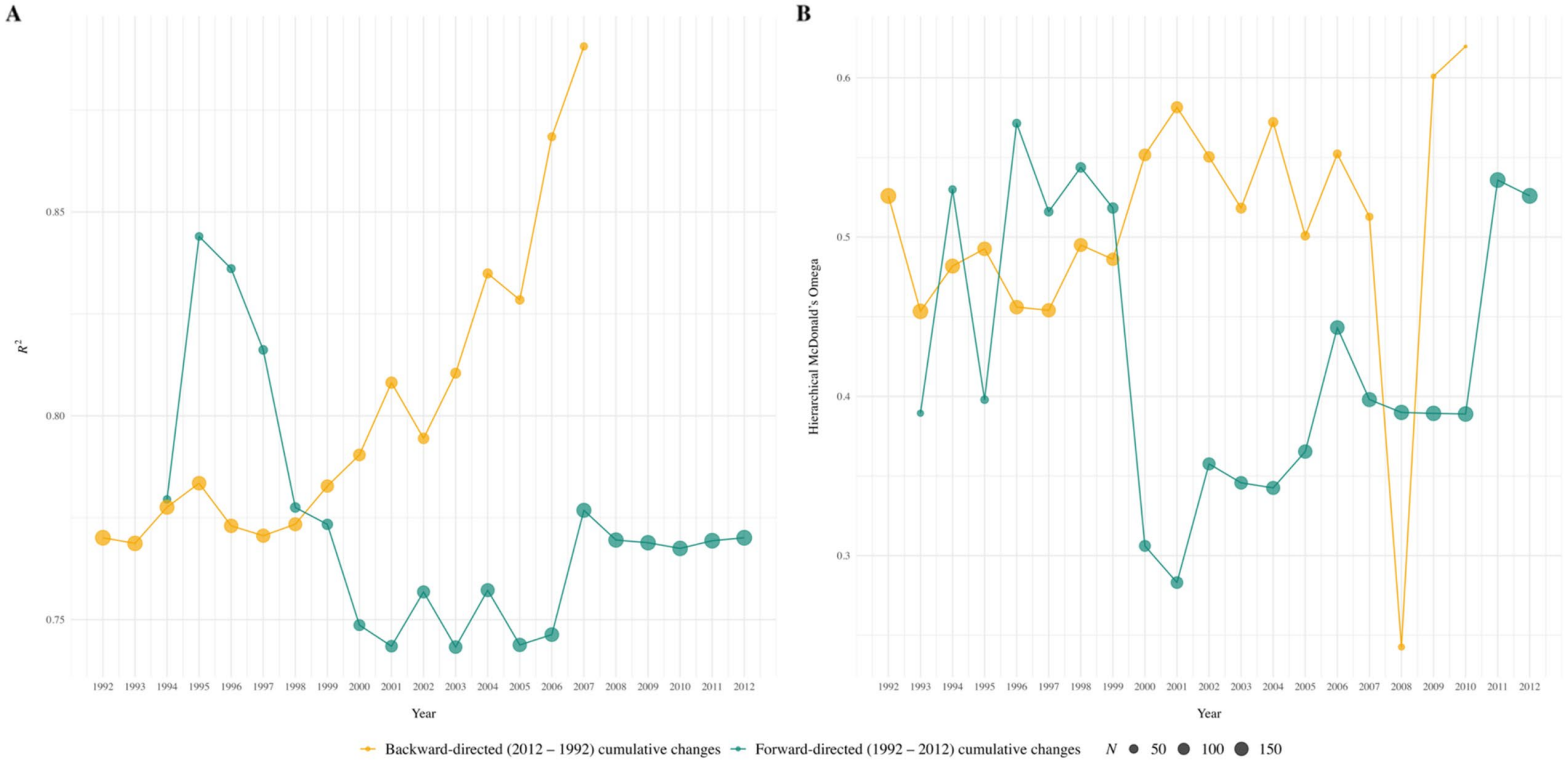
Forward-directed (1992 to 2012) and backward-directed (2012 to 1992) cumulative cross-temporal changes in *R*^2^ **(Panel A)** and McDonald’s ω_h_ **(Panel B)** across ten WIT subscales. The cumulation of those years that contained data from at least ten participants was chosen as the starting point; within backward-directed factor analyses for 2012 to 2008, 2012 to 2009, and 2012 to 2010, the model did not converge due to low case numbers.

**Figure 4:**
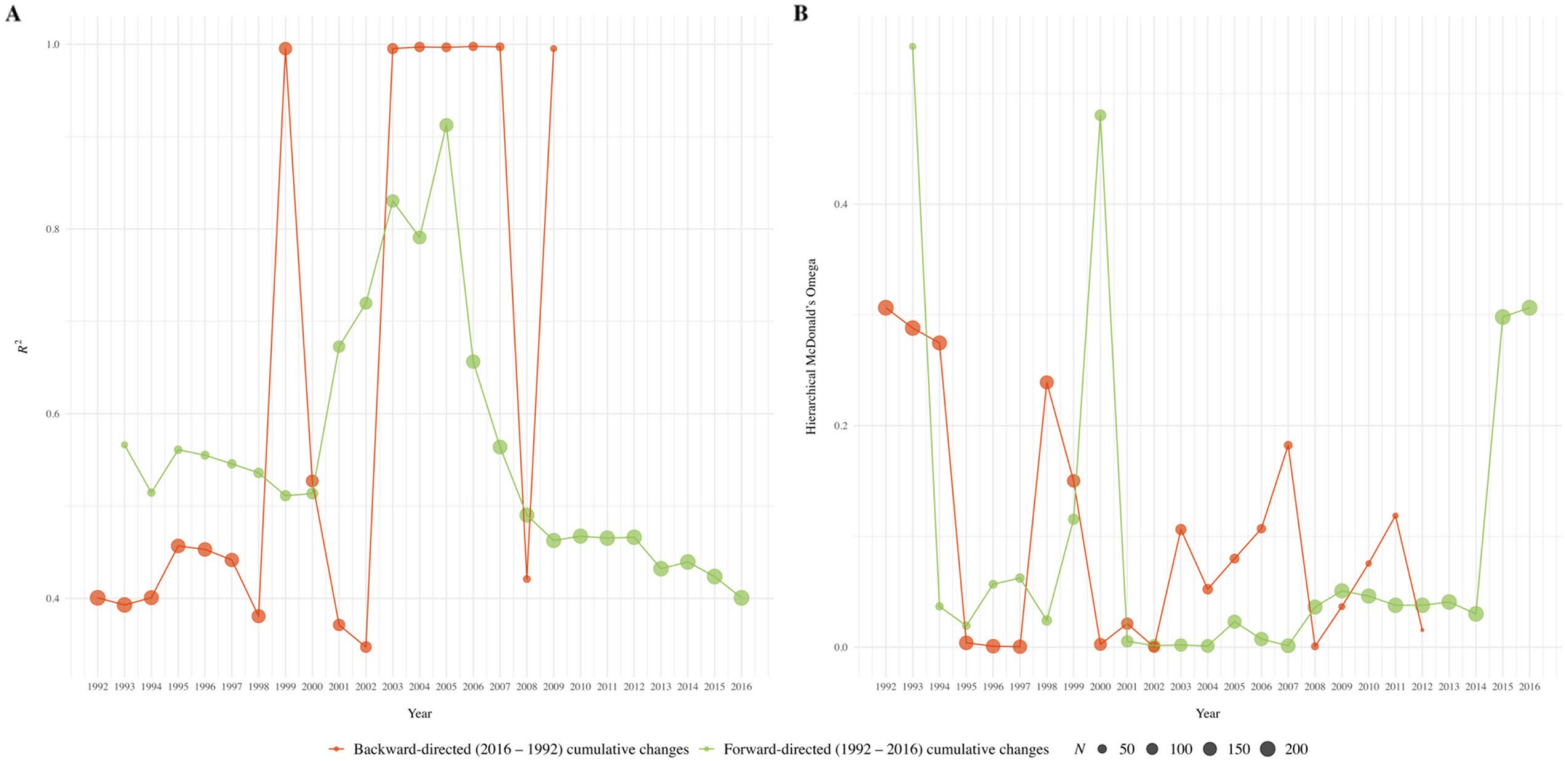
Forward-directed (1992 to 2016) and backward-directed (2016 to 1992) cumulative cross-temporal changes in *R*^2^ **(Panel A)** and McDonald’s ω_h_ **(Panel B)** across three WIT subscales. The cumulation of those years that contained data from at least ten participants was chosen as the starting point; within backward-directed factor analyses for 2016 to 2010, 2011 to 2009, and 2016 to 2012, models did not converge due to low case numbers.

Our analyses of cross-temporal changes in the positive manifold of intelligence revealed no systematic changes in *R*^2^ values (i.e., in the explained variance of psychometric g) across any subset of recruitment years or the number of included subtests (see Figures 2–4). Similarly, no consistent pattern emerged in the trajectory of McDonald’s ω_h_ over time for any subset, indicating no systematic changes in the *g* saturation of the WIT. Cumulative forward and backward analyses of the respective subsets further supported these findings of no substantial or systematic changes in either *R*^2^ or ω_h_. Instead, nonparallel trends in the respective graphs suggest that cross-temporal changes in numerical values may reflect a function of variations in sample sizes, rather than genuine changes in the positive manifold of intelligence. Numerical results for all subsets of recruitment years are detailed in Tables S1 to S3 (https://osf.io/3pnwj/).

Finally, ability ranges showed inconsistent cross-temporal change patterns, yielding both positive and negative correlations, thus indicating no evidence for systematic changes in the strength of the positive manifold of intelligence over time (β range: −0.14 to 0.34 *p* range: < 0.001 to 0.11).

## 4 Discussion

Here, we provide a formal investigation of cross-temporal changes in the positive manifold of intelligence among a very specific sample of Austrian Air Force pilots and air traffic controllers. Our analyses revealed substantial positive Flynn effects in eleven out of twelve subscales from 1992 to 2016. However, we found no evidence of a cross-temporally decreasing strength of the positive manifold of intelligence within these participants. This presents several points of interest, as discussed below.

We observed IQ test score gains across virtually all subtests of the WIT over a period of 25 years. During this timeframe, we found no changes in the strength of the positive manifold and the intercorrelations among these subtests. These findings contrast recent results of systematic cross-temporal decreases in the positive manifold of intelligence in population-representative Austrian samples over the past 20 years (Oberleiter et al., 2024a) and achievement-*g* decreases in Italian students (Pietschnig et al., 2023). However, it was to be expected that changes in the positive manifold would behave differently in our participants because the present sample is highly specific. It was selected according to the very strict criteria of the Austrian Armed Forces, performed above the population mean across all investigated domains, and can therefore be classified as belonging to the upper tail of the ability distribution. Psychometric *g* is typically assumed to have less predictive value in higher-ability individuals (namely, due to SLODR). Furthermore, due to the restricted range of test scores in this higher-ability sample, the IQ variance is smaller than in the general population. This restriction can attenuate the strength of observed correlations among subtests and, as a result, reduce the detectability of changes in the positive manifold of intelligence over time. This assumption is reasonable because cognitive high performers are likely to be more specialized than general population samples. Accordingly, any changes in the positive manifold of intelligence or *g* should be more challenging to detect in high-ability samples because it can be assumed that they have a comparatively low *g* saturation which, in turn, potentially leads to a ceiling effect with respect to cross-temporal *g* changes.

In terms of our decathlete analogy, among those who specialize in running and excel in that domain, fine-grained differences may begin to emerge as their results cluster narrowly at the upper tail of the ability distribution. At this level, overall decathlon performance, akin to what we introduced as “athletic *g*,” no longer determines the incremental advantages among specialized runners. Instead, distinctions between these high achievers might stem from nuances in their specialized training, techniques, and domain-specific adaptations. In other words, while *g* is fundamental in a decathlete’s overall performance, its influence could diminish when distinguishing between cognitive high achievers who excel in a specific cognitive domain. This mechanism may account for the observed inconsistencies in the trajectories of the Flynn effect in recent reports (Oberleiter et al., 2024a; Andrzejewski et al., 2025).

We observed no cross-temporal changes in the ability range among Austrian Air Force pilots and air traffic controllers. This supports the idea that their ability profiles may be cross-temporally stable, leaning more toward asymmetry (i.e., lower *g* saturation) than symmetry (i.e., higher *g* saturation). In this vein, they may be considered to be already specialized in specific domains, and therefore, no substantial decline of the positive manifold of intelligence and intercorrelations of intelligence subdomains over time are to be expected.

Our observation of no systematic changes in the positive manifold in this very specific sample conforms to the idea of differential trajectories of IQ and domain intercorrelation changes in different ability segments (Flynn and Shayer, 2018; Oberleiter et al., 2024a). However, the substantial Flynn effects in most of the domains examined here contrast with the idea of a test score gain stagnation in above-average performing individuals.

In terms of test score gains, we observed the strongest Flynn effects for verbal arithmetic tasks. Verbal arithmetic can be considered to represent quantitative reasoning which maps on the stratum II domain fluid reasoning (*g*_f_) within the CHC model (Schneider and McGrew, 2018). Similarly, the other *g*_f_-related subscales (letter sequencing, observation and processing, and spatial reasoning) yielded positive and mostly significant Flynn effects. Consequently, our results fit well with prior observations of larger gains in fluid compared to crystallized IQ domains (Pietschnig and Voracek, 2015).

In addition to its association with fluid reasoning (*g*_f_), verbal arithmetic traditionally demonstrated a high loading on the stratum II domain of quantitative knowledge (*g*_c_). Here, *g*_c_ was further assessed using two subscales (namely, numerical reasoning and number sequencing) of which both showed significant test score increases in our sample as well. This is consistent with findings of positive Flynn effects on this domain in a prior study (Lazaridis et al., 2022), although not all examined *g*_q_-related subtests yielded directionally consistent evidence in this account.

Subscales related to working memory were consistently positive as well, although medium-term memory showed considerably larger gains than digit span performance. This contrasts evidence of decreasing working memory performance in the Austrian general population from the early 2000s to the mid-2010s (Lazaridis et al., 2022), thus further suggesting differentiation of the Flynn effect on the stratum I level.

We observed a positive Flynn effect in the verbal fluency subscale of the stratum II domain retrieval. To our knowledge, this represents the first account for a Flynn effect in a test that assesses ease of word production.

In the present study, the stratum II domain comprehension knowledge was assessed using three subscales. While proverb comprehension showed a positive (albeit non-significant) Flynn effect, semantic similarities showed virtually no test score changes, and verbal analogies yielded a significant negative Flynn effect. This ambiguous pattern of results in the *g*_c_ domain may indicate once again a differentiation on the stratum I level, particularly because prior evidence from Germanophone comprehension knowledge changes in the 2000s showed virtually ubiquitous positive Flynn effects (e.g., Lazaridis et al., 2022; Pietschnig et al., 2010, 2011; Oberleiter et al., 2024a).

However, the comparatively large number of substantial positive Flynn effects across several domains in our present study seems remarkable because previous research typically has found that samples from the upper segments of the population ability distribution exhibit less substantial gains than those from the lower segments (Flynn and Shayer, 2018; Oberleiter et al., 2024a).

### 4.1 Limitations

Some limitations need to be acknowledged when interpreting the results of this study. First, only candidates who had been administered the WIT were included in the analyses, in order to ensure comparability of results. Candidates who had been administered other test instruments were excluded. As a result, our dataset includes only candidates who had been examined until 2016.

Second, in this study, we investigated a highly specific sample of Austrian Air Force pilots and air traffic controllers, comprising 97% men. Consequently, we could not assess potential differences in change trajectories according to sex. However, past targeted investigations of sex differences on the Flynn effect indicated no meaningful differences between men and women (Pietschnig et al., 2011), thus suggesting similar trajectories for cognitively high-performing women.

Third, change estimates based on a limited number of participants may lead to numerical volatility of effects (Schönbrodt and Perugini, 2013). Consequently, whilst the sign of changes may be confidently interpreted, we caution against interpreting the magnitude of some remarkably large changes per decade, such as those for the letter sequencing subtest, at face value. However, our comparatively small sample is due to its highly select and specific nature (namely, Austrian Air Force pilots and air traffic controllers). The presently analyzed dataset comprises only individuals who successfully completed the highly selective aptitude assessments for Austrian military pilots and air traffic controllers across 25 years. As such, it reflects a near-complete record of those who met the stringent selection criteria of the Austrian Armed Forces within this time frame, making it a particularly informative and relevant sample for the present research question.

Fourth, due to the unavailability of item-level data, we were unable to assess measurement invariance of the WIT across the respective testing time points. Such analyses (e.g., Pietschnig et al., 2013) are important to disentangle genuine changes in cognitive abilities from those potentially driven by item drifts. However, prior related evidence suggests that intelligence subtests assessing abilities other than crystallized intelligence, such as fluid intelligence (*g*_f_), quantitative knowledge (*g*_q_), or working memory (*g*_wm_), are less likely to be influenced by cross-temporal differential item functioning and more likely to reflect genuine test score changes (e.g., Lazaridis et al., 2022; Oberleiter et al., 2024a, 2024b).

## 5 Conclusion

In all, we show a predominantly positive and substantial Flynn effect in a sample of Austrian Air Force pilots and air traffic controllers. Our results showed no evidence for an increasing ability differentiation as a driver of changing test score patterns, conceivably owing to the expectable considerable specialization of the sample investigated here. Ambiguity in change patterns in the subscales relating to the CHC stratum II domains of working memory and comprehension knowledge suggest stratum I-based differentiation of the Flynn effect in these domains.

## Data Availability

The data analyzed in this study is subject to the following licenses/restrictions: due to legal restrictions, the archival data provided by the Austrian Armed Forces are not publicly available. Requests to access these datasets should be directed to Jakob Pietschnig, jakob.pietschnig@univie.ac.at.
